# A novel prion protein variant in a patient with semantic dementia

**DOI:** 10.1136/jnnp-2017-315577

**Published:** 2017-06-01

**Authors:** Joanna Kenny, Ione Woollacott, Carolin Koriath, Laszlo Hosszu, Gary Adamson, Peter Rudge, Martin N Rossor, John Collinge, Jonathan D Rohrer, Simon Mead

**Affiliations:** 1 MRC Prion Unit, Department of Neurodegenerative Disease, UCL Institute of Neurology, London, UK; 2 NHS National Prion Clinic, National Hospital for Neurology and Neurosurgery, University College London Hospitals NHS Foundation Trust, London, UK; 3 Department of Neurodegenerative Disease, UCL Institute of Neurology, London, UK

**Keywords:** CREUTZFELDT-JAKOB DISEASE, DEMENTIA, NEUROGENETICS, PRION, SEMANTIC DEMENTIA

Prion diseases are a group of fatal neurodegenerative diseases that can be sporadic, inherited or acquired. Inherited prion diseases are caused by mutations in the prion protein gene, *PRNP*, usually single nucleotide substitutions or structural variants of an octapeptide repeat encoding region. Although the classical presentation of sporadic Creutzfeldt Jakob disease (CJD) is rapidly progressive ataxia, myoclonus and cognitive decline, the presentation of genetic cases is variable and decline can be much slower.

Prion diseases can mimic many neurodegenerative diseases. Genetic dementias are pleiotropic, with similar clinical syndromes being caused by mutations in different genes; additionally, mutations in a single gene may cause diverse clinical phenotypes. Due to the diagnostic difficulties arising from this clinical and genetic heterogeneity, next-generation sequencing technologies including gene panels and exome sequencing are helpful in elucidating genetic causes of dementia.^1^

Here we report a novel variant in *PRNP* in a patient diagnosed with semantic dementia, a variant of frontotemporal dementia (FTD). Patients with semantic dementia develop progressive loss of semantic knowledge resulting in significant early language impairment, with subsequent wider cognitive impairment and behavioural problems. MRI appearances are characteristic with focal asymmetric anteroinferior temporal lobe atrophy. The predominant histopathological finding is accumulation of Tar DNA-binding protein 43 (TDP-43) deposits in the temporal and frontal lobes, although other pathologies, including Alzheimer’s disease, are occasionally observed.^2^ A Mendelian genetic aetiology is rarely documented.

A woman in her seventh decade presented with a 1-year history of rapidly progressive language problems and behavioural change. She had particular difficulty naming objects and suffered from impaired comprehension, but had preserved recognition of faces and episodic memory. Over the same time period her personality changed, becoming disinhibited and exhibiting obsessional behaviour. She also struggled with cooking but not with self-care or housework. She had no significant surgical or medical history, apart from a 5-year history of tinnitus and deafness, and took no regular medications.

A family history of dementia was known: her mother developed dementia of uncertain aetiology in her seventh decade, which progressed steadily with a total duration of 10 years. Her father died in his eighth decade of a myocardial infarction. There was no other relevant family history.

Physical examination revealed bilateral sensorineural hearing loss, globally brisk tendon reflexes, positive pout reflex and mild bilateral limb apraxia, but was otherwise unremarkable. She scored 23/30 on Mini-Mental State Examination (MMSE). On formal neuropsychometric testing, she obtained a verbal IQ of 66 and performance IQ of 78. Testing of specific cognitive domains revealed significantly impaired single word comprehension (Peabody Picture Vocabulary Test) and anomia (Oldfield Naming Test), as well as a surface dyslexia and dysgraphia, consistent with an impairment of verbal semantic knowledge. Other cognitive domains, including calculation, non-verbal reasoning (Raven’s Coloured Matrices), episodic memory (Camden Pictorial Recognition Memory Test) and visuospatial skills, were intact.

Dementia screening bloods and cerebrospinal fluid analysis (undertaken prior to the availability of Aβ42 and tau measurements) were normal. An MRI scan was reported as showing scattered white matter lesions in both cerebral hemispheres, presumed secondary to mild small vessel disease, with evidence of generalised brain atrophy, although the scan was unavailable for review.

A diagnosis of semantic dementia was made based on the history and pattern of her cognitive deficits.

She was next reviewed 5 years later when she had deteriorated significantly with very limited comprehension and frequent use of jargon words, as well as presenting with visual agnosia. Her behaviour had deteriorated, becoming severely apathetic, losing interest in all of her previous hobbies, increasingly obsessive, particularly with time-keeping, markedly disinhibited, and developing a sweet tooth. She could no longer do housework and struggled with personal activities of daily living.

The patient was subsequently lost to follow-up and died 10 years after onset without being examined at postmortem.

Twenty years after presentation, a DNA sample was tested using the Medical Research Council (MRC) Dementia Gene Panel, a custom next-generation sequencing technology covering 17 dementia genes, including common FTD-associated genes such as *MAPT* and *GRN*.^1^ Samples were tested separately for *C9orf72* expansions and *PRNP* octapeptide repeat insertions/deletions. Results were analysed for known and novel variants and confirmed by Sanger sequencing.

**Figure 1:**
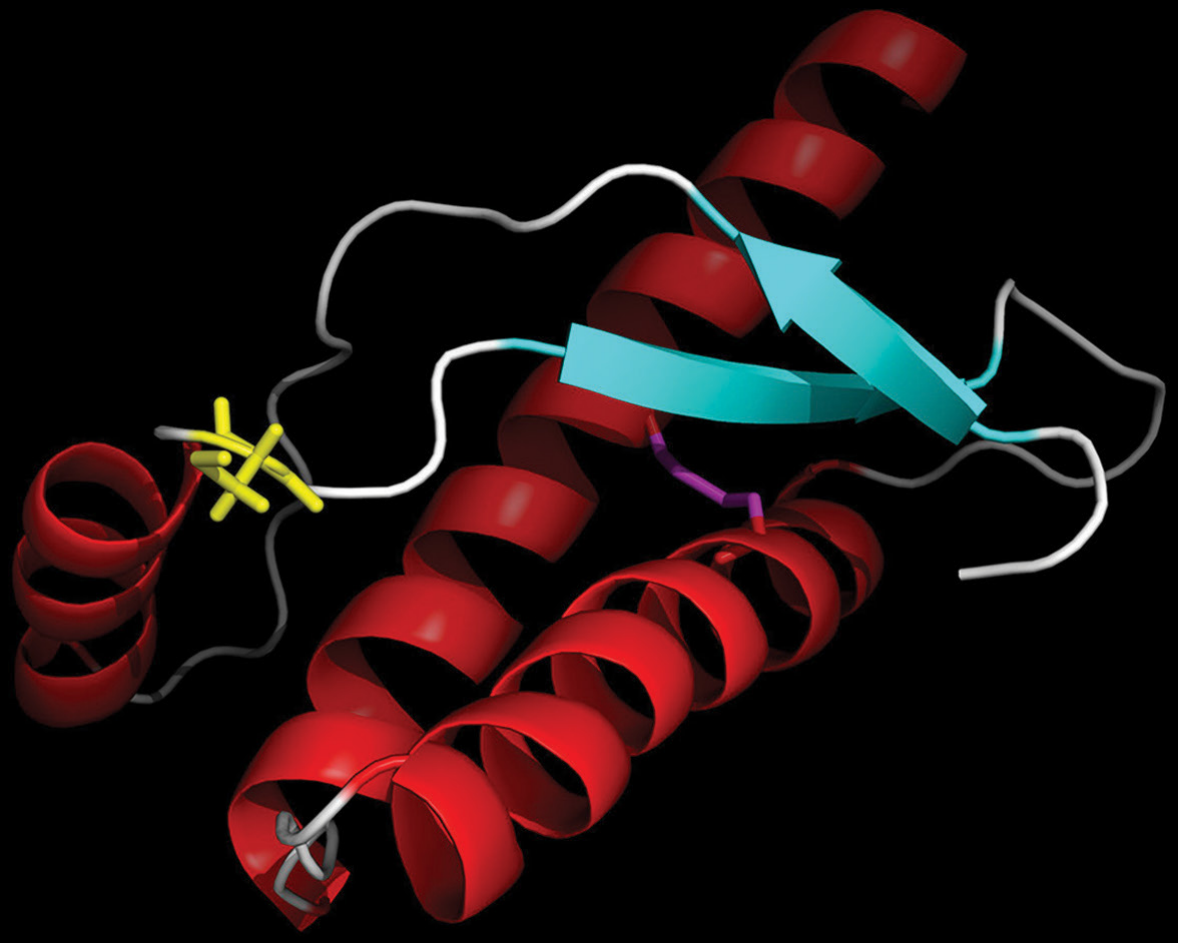
Ribbon representation of the structure of the folded domain of the human prion protein,^3^ with α-helices coloured red, β-strands cyan and loops white. The mutated cysteine side chain of residue 156 is shown in yellow. The disulphide bond linking the side chains of the other cysteines in the protein (Residues 179 and 214) is shown in magenta. This figure was prepared using PyMOL (Schrödinger).

We discovered a novel *PRNP* variant (R156C, g.4680332C>T), which is absent from 141 362 unrelated individuals’ exome and whole-genome sequences collated by the Exome Aggregation Consortium (http://gnomad.broadinstitute.org/). The patient was homozygous for methionine at codon 129 (129MM). No other pathogenic mutations or risk factors for neurodegeneration were identified. The variant results in the replacement of a large, positively charged arginine residue, located immediately after the first α-helix of the prion protein (PrP^C^), with a smaller polar, uncharged cysteine (figure 1). As this amino acid is located on the PrP^C^ surface, it is unlikely to cause a gross conformational change in PrP^C^. However, cysteines may form intermolecular or intramolecular covalent disulphide bonds. Indeed, the two wild-type cysteines present in PrP^C^ form a disulphide bond, which is essential to the fold stability of the protein.^3^ We hypothesise that the introduction of an additional cysteine might disrupt the normal PrP^C^ disulphide bond or result in the formation of intermolecular disulphide bonds, as observed previously.^4^ A similar arginine to cysteine mutation, R208C, has been found in a single case with CJD.^5^ A few other mutations have been reported in the region between first and second α-helices including Q160X, Y163X, D167G and D167N, some of which follow a very atypical clinical course.^6^ No mutations have been reported at position 156, but in silico modelling (Polyphen, SIFT, FATHMM, MutationAssessor, MutationTaster) suggests that this variant is likely to be pathogenic. However, Minikel *et al* 7 demonstrated that the frequency of *PRNP* missense and truncating variants in the general population is incompatible with the incidence of prion diseases. Novel variants thus need to be assessed with caution regarding their pathogenicity in the absence of segregation or functional data despite their rarity.

We report a novel missense mutation of *PRNP* in a patient with semantic dementia, which would at present be classified as likely pathogenic. Patients with semantic dementia would not normally be recommended for genetic studies, particularly of *PRNP*. The use of next-generation sequencing technologies will be increasingly important in elucidating the causes of pleiotropic conditions such as familial dementia.
